# Scalloped tongue: an additional, accessible and useful tool to detect severe obstructive sleep apnea?

**DOI:** 10.3389/frsle.2025.1652532

**Published:** 2025-08-20

**Authors:** Francisca Nieto Guimarães, Joana Canadas, Maria Gonçalves Cunha, Vera Durão, Paula Rosa, Marcelo Rabahi, Ding Zou, Flávio Magalhães da Silveira

**Affiliations:** ^1^Pulmonology Department, Vila Franca de Xira Hospital, Vila Franca de Xira, Portugal; ^2^Faculty of Medicine and Graduate Program in Health Sciences at the Federal University of Goiás, Goiânia, Brazil; ^3^Center for Sleep and Vigilance Disorders, University of Gothenburg, Gothenburg, Sweden; ^4^SleepLab Rio de Janeiro, Rio de Janeiro, Brazil

**Keywords:** scalloped tongue, severe OSA (obstructive sleep apnea), hypoxic burden, diagnosis, primary care

## Abstract

The anatomy of the upper airway can influence the risk of obstructive sleep apnea (OSA). However, there is limited evidence supporting the link between scalloped tongue (ST) and nocturnal intermittent hypoxia. This study aimed to investigate if ST could serve as a clinical indicator of OSA, particularly severe OSA. Over a 4-month period from October 2023 to January 2024, 160 patients underwent level 1 polysomnography at a sleep laboratory in Brazil. Demographics, body mass index (BMI), neck circumference (NC), presence of ST, Epworth Sleepiness Scale score, apnea hypopnea index, oxygen desaturation index (ODI) and time under 90% of oxygen saturation were included in a database. Logistic and multiple linear regression models were performed. A *p-*value <0.05 was considered as the lower threshold of significance. Most (90%) patients had OSA, 41% classified as severe. Older age and a wider NC significantly increased the risk of OSA. Older age, higher BMI, wider NC, and ST significantly increased the risk of severe OSA, and there was a statistically significant positive correlation between the presence of ST and ODI (*p* = 0.001). The presence of ST increased ODI by 6.723/h, adjusted for age, BMI, and NC. The combined presence of NC ≥ 40 cm and ST significantly increased the risk of severe OSA (OR 4.210, *p* < 0.001), and significantly impacted ODI estimates. Incorporating tongue and NC assessment in OSA screening, both objective and easily observable clinical signs, may help physicians in the prompt identification of severe cases that benefit from early positive airway pressure therapy.

## Background

Obstructive sleep apnea (OSA), a condition with an overall prevalence ranging from 9% to 38% in the general adult population, is characterized by repeated episodes of partial or total airway obstruction during sleep (Senaratna et al., 2017). Although very common, it is often underdiagnosed (Patel, 2019). Primary care physicians are crucial in OSA screening, as they are typically the first point of contact for patients and play a key role in recognizing its clinical features, initiating appropriate referrals for diagnostic confirmation, and facilitating access to specialized care (Lieberman, 2009). The diagnosis can be challenging, as many OSA patients have a subjective perception of good sleep or are unaware if they snore (Nam et al., 2016). Older age, obesity, neck circumference (NC) and the structure of the throat are well known signs associated with an increased risk of OSA, which can help the physician (Senaratna et al., 2017; Chung et al., 2016; Neelapu et al., 2017).

The tongue plays a significant role in regulating the size and shape of the upper airway (Kim et al., 2014; Baker et al., 2025). Fat accumulation at the base of the tongue, may alter its morphology, narrow the retroglossal airway and increase the risk of OSA (Kim et al., 2014; Baker et al., 2025). However, other tongue and oral or cranio-facial factors can also contribute to a narrowed airway. Macroglossia, for instance, is considered a risk factor for OSA and is correlated with increased severity (Baker et al., 2025).

The interplay between the tongue and the surrounding anatomical structures can lead to the development of clinical signs that can be useful and rapidly identified.

**Figure 1:**
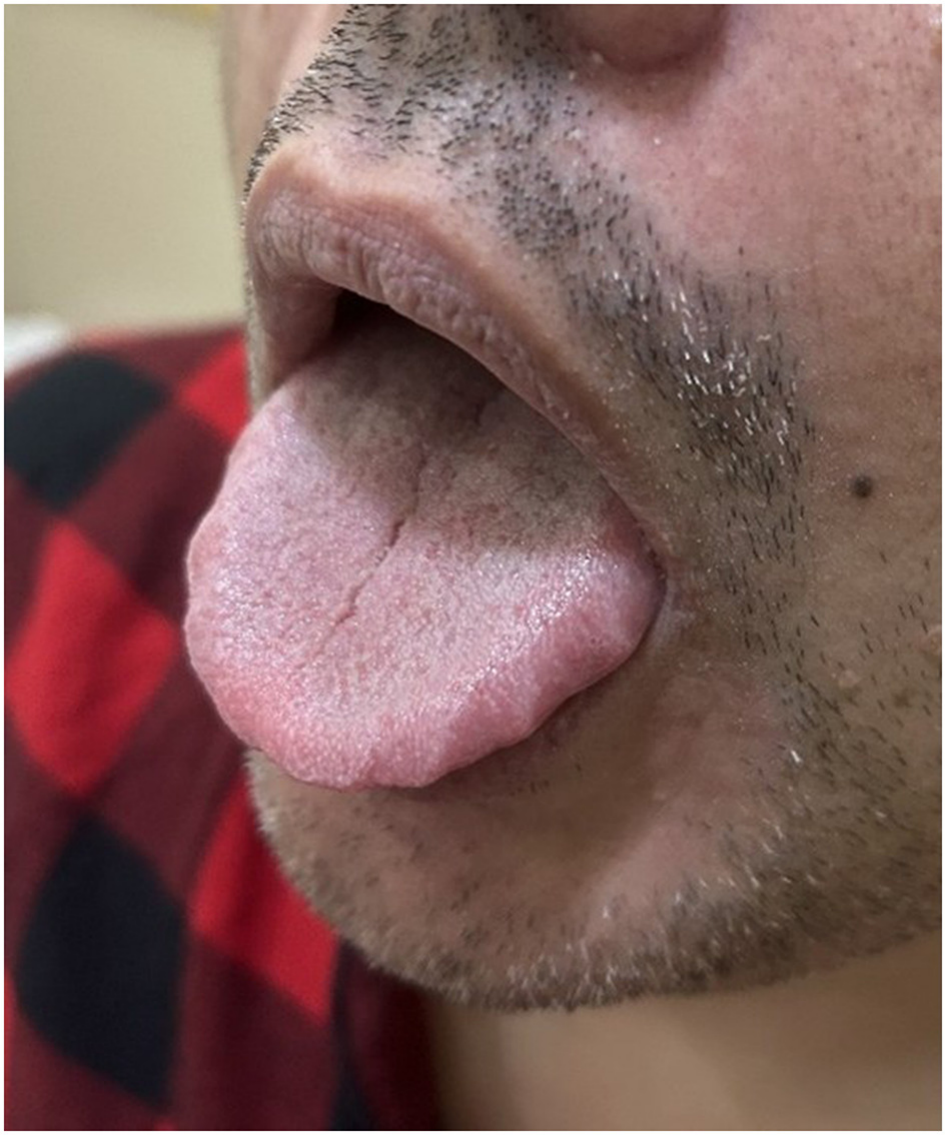
Patient with ST evidence during tongue protrusion included in this study. Written informed consent for publication of the photograph was obtained and recorded.

Tongue indentations along the lateral borders of the tongue, or scalloped tongue (ST) (Figure 1), have been closely related to tongue width and volume (Tomooka et al., 2017; Yanagisawa et al., 2007; Hata and Ihara, 2024; Scoppa, 2019). However, they can also derive from other factors, including tongue thrusting and dental alterations/deformities (Scoppa, 2019).

Some limited evidence has emerged supporting the link between ST and nocturnal intermittent hypoxia or OSA (Tomooka et al., 2017; Weiss et al., 2005). Weiss TM et al. described an association between ST and OSA in a small sample (Weiss et al., 2005). Tomooka K et al., on the other hand, were not able to establish a correlation between the presence of ST and OSA or nocturnal intermittent hypoxia (Tomooka et al., 2017).

This study aimed to investigate if ST could serve as an additional clinical indicator of OSA, particularly severe OSA.

## Methods

### Study population

Over a 4-month period from October 2023 to January 2024, 525 patients underwent level 1 polysomnography (PSG) at a sleep laboratory in Rio de Janeiro. Patients undergoing OSA treatment (*n* = 27) were excluded. All patients underwent tongue and teeth examination. No patients had tongue tie. Patients with incomplete denture were excluded (*n* = 22). Other craniofacial features were not assessed. Patients who did not consent to have their tongue photographed were excluded (*n* = 156). Patients with fewer than two photographs taken by the technician or those with one or both photographs with insufficient quality to assess both sides of the tongue were excluded (*n* = 246). Patients with incomplete clinical data were also excluded (*n* = 74). In total, 160 patients were eligible for further analyses.

### Clinical data

On the evening of the PSG, experienced sleep technicians registered the following clinical data: gender, age, body mass index (BMI), NC, presence of ST, and Epworth Sleepiness Scale (ESS) score. NC was measured in centimeters (cm) using a flexible tape at the level of the laryngeal prominence, with participants in the standing position, head held erect. Excessive daytime sleepiness (EDS) was subjectively assessed using the ESS whereby a score ≥11 (from 0 to 24) was deemed as indicative of excessive daytime sleepiness.

### Assessment of scalloped tongue

All participants were instructed to protrude and relax their tongues. A team of experienced sleep technicians took two photographs of each tongue (including the left and right side) using an iPhone 11 camera. Using these images, three trained physicians independently assessed the presence or absence of ST in a binary manner, recording it as either “present” or “absent.” The condition was defined by the presence of at least two indentations on both lateral borders of the tongue, regardless of severity. In cases of disagreement, the final classification was based on majority decision (2 out of 3 physicians). This data collection was conducted prior to PSG results.

### Polysomnography

PSG were performed at a sleep laboratory in Rio de Janeiro, Brazil: SleepLab—*Laboratório de Estudo dos Distúrbios do Sono*. All participants underwent a full PSG using *Somnologica Embla*^®^*, Alice PDx Philips*^®^ *and NeuroVirtual* systems, which included continuous recording of electroencephalography, electrooculography, electromyography (chin and legs), electrocardiography, airflow, chest and abdominal impedance belts, oxygen saturation, snoring microphone, and body position sensors. Polysomnographic data were manually scored according to the 2017 American Academy of Sleep Medicine statement criteria (Berry et al., 2012) by two board-certified sleep physicians, who were blind to the presence or absence of a ST.

Obstructive apneas were defined as a ≥90% reduction in airflow from baseline, lasting for at least 10 s, associated with persistent respiratory effort (Berry et al., 2012). Hypopneas were classified as a ≥30% reduction of the airflow for at least 10 s associated with ≥3% oxygen desaturation or arousal (Berry et al., 2012). The apnea–hypopnea index (AHI) was calculated as the sum of apnea and hypopnea episodes per hour of sleep (Berry et al., 2012). The polysomnographic diagnosis of OSA was based on a AHI ≥5.0/h and the classification of severe OSA was based on a AHI ≥30.0/h (Berry et al., 2012). Oxygen desaturation index (ODI) was defined as the number of ≥3% arterial oxygen desaturations per hour of sleep (Berry et al., 2012).

### Statistical analysis

Demographics (age, gender), BMI (kg/m^2^), NC (cm), presence of ST, ESS score, AHI (events/hour), ODI (events/hour) and percentage of time spent with oxygen saturation below 90% (T90) were included in a database. OSA and severe OSA were coded as binary variables. Independent logistic regression models were performed to evaluate the risk of OSA (AHI ≥ 5/h) and severe OSA (AHI ≥ 30/h), using the independent variables: age, gender, BMI, NC, ST and ESS score. For severe OSA, a second logistic regression model was performed, which included as independent variables: age, gender, BMI, ESS score, and NC ≥ 40 cm combined with ST codified as a single binary variable considering the presence or absence of these two features simultaneously. Final models were obtained after successive exclusion of the most non-significant variable, and the AUC was determined for each model. Multiple linear regression models were performed to test associations between ODI and the independent variables: age, gender, NC, weight, BMI, ST and ESS score.

A *p-*value <0.05 was considered as the lower threshold of significance. Descriptive statistics were used for the basic analysis of data. All analyses were conducted using the software EXCEL (Microsoft 365) and IBM SPSS Statistics 28.

## Results

Most patients were men (*n* = 93; 58.1%), the mean age was 48.8 years old, and the mean BMI was 30.9 Kg/m^2^. Eighty-one (50.6%) patients had ST. Most (90%) had OSA, 41% of whom were classified as severe. Their clinical characteristics and polysomnogram results are summarized in Table 1. Older age and a wider NC significantly increased the risk of OSA (OR 1.072, *p* = 0.005; and 1.631, *p* < 0.001, respectively). There was no significant association between the presence of ST and OSA (*p* = 0.160) (Table 2). Older age, higher BMI, wider NC, and ST significantly increased the risk of severe OSA (OR 1.056, *p* < 0.001; 1.077, *p* = 0.035; 1.151, *p* = 0.016; and 3.065, *p* = 0.004, respectively). The combined presence of NC ≥ 40 cm and ST significantly increased the risk of severe OSA (OR 4.210, *p* < 0.001) (Table 2).

**Table 1:** Demographics and characteristics of the patients included in the study.

**Patient's characteristics**	**Total (*n =* 160)**	**OSA (*n =* 144)**	**Mild/moderate OSA (*n =* 85)**	**Severe OSA (*n =* 59)**	***p*-value**
Age, mean ± SD	49 ± 15	50.2 ± 13.8	48.2 ± 13.5	53.1 ± 13.8	0.033
Male gender, *n* (%)	93 (58.1)	89 (61.8)	48 (56.5)	41 (69.5)	0.114
Weight (Kg), mean ± SD	89.9 ± 21.3	91.9 ± 21.3	88.7 ± 20.7	96.6 ± 21.5	0.029
BMI (Kg/m^2^), mean ± SD	30.9 ± 6.4	31.4 ± 6.5	30.2 ± 6.4	33.1 ± 6.3	0.010
NC (cm), mean ± SD	39.7 ± 4.2	40.3 ± 3.8	39.5 ± 3.7	41.5 ± 3.7	0.002
ESS score, mean ± SD	10 ± 5	10 ± 5	9.2 ± 4.9	10.4 ± 5.1	0.177
Patients with ST, *n* (%)	81 (50.6)	77 (53.5)	38 (44.7)	39 (66.1)	0.011
AHI (events/hour)	27.3 ± 22.4	30.1 ± 21.9	15.9 ± 7.0	50.5 ± 19.9	<0.001
ODI (events/hour)	16.0 ± 20.7	17.7 ± 21.1	6.4 ± 5.3	34.0 ± 24.6	<0.001
T90 (%)	0.1 ± 0.2	0.1 ± 0.2	0.0 ± 0.1	0.2 ± 0.2	<0.001

p-value refers to comparison of the groups: Severe OSA vs. Mild and Moderate OSA.

OSA, obstructive sleep apnea (AHI ≥ 5/h) and severe OSA (AHI ≥ 30/h); BMI, body mass, index; NC, neck circumference in centimeters; ESS, Epworth Sleep Scale; ST, scalloped tongue; AHI, apnea-hypopnea index; ODI, oxygen desaturation index; T90, percentage of time spent with oxygen saturation below 90%.

**Table 2:** Risk of obstructive sleep apnea (OSA) and of Severe OSA.

**Variables included in the models**	OR^*^[95% CI]	***p*-value**
**OSA** ^*^		
Age (years)	1.072 [1.021–1.126]	0.005
Neck circumference (cm)	1.631 [1.288–2.067]	<0.001
Scalloped tongue	2.821 [0.663–11.999]	0.160
**Severe OSA (Model 1)** ^**^		
Age (years)	1.056 [1.025–1.088]	<0.001
BMI (Kg/m^2^)	1.077 [1.005–1.153]	0.035
Neck circumference (cm)	1.151 [1.026–1.290]	0.016
Scalloped tongue	3.065 [1.425–6.593]	0.004
**Severe OSA (Model 2)** ^***^		
Age (years)	1.054 [1.025–1.084]	<0.001
BMI (Kg/m^2^)	1.092 [1.028–1.161]	0.004
Neck circumference ≥40cm + Scalloped tongue	4.210 [1.928–9.193]	<0.001

OR, odds ratio; 95% CI, confidence interval of 95%; BMI, body mass index.

^*^Final logistic regression model for OSA after successive exclusion of the most non-significant variable, namely: Epworth score (*p* = 0.99), female gender (*p* = 0.973), weight (*p* = 0.266), and BMI (*p* = 0.381). Hosmer and Lemeshow Test *p* = 0.845, AUC = 0.942 [0.904–0.980].

^**^Final logistic regression model 1 for Severe OSA after successive exclusion of the most non-significant variable: Epworth score (*p* = 0.344), female gender (*p* = 0.168), and weight (*p* = 0.118). Hosmer and Lemeshow Test *p* = 0.788 AUC = 0.747 [0.666–0.829].

^***^Final logistic regression model 2 for Severe OSA including combined neck circumference (NC) **≥40 cm and scalloped tongue (ST)**, and after successive exclusion of the most non-significant variable: Epworth score (*p* = 0.229), weight (*p* = 0.77), and female gender (*p* = 0.105). Hosmer and Lemeshow Test *p* = 0.598, AUC = 0.776 [0.701–0.851].

There was a statistically significant positive correlation between age, weight, BMI, NC, ST, and ODI [correlation coefficients of Spearman (rho) = 0.288, 0.444, 0.375, 0.551 and 0.253, respectively, all with *p* ≤ 0.001]. Female gender had a significant negative correlation with ODI (rho= −0.358, *p* < 0.001). Gender was significantly correlated to ST and NC, while weight was significantly associated with age, gender, BMI, and NC, so gender and weight were excluded from multiple linear regression models for estimated ODI to avoid multicollinearity. Age, BMI, NC, and tongue indentation had a significant impact on ODI estimates. The presence of ST increased ODI by 6.723/h, adjusted for age, BMI, and NC (Table 3). The combined presence of NC ≥ 40cm and ST also had a significant impact on ODI estimates, increasing it by 7.557/h, adjusted for age and BMI. There was no statistically significant correlation between ST and T90 (*p* = 0.126).

**Table 3:** Multiple Linear Regression models for estimated ODI (dependent variable).

**Variables included in the models**	β^*^[95% CI]	***p*-value**
**ODI (events/hour) Model 1** ^*^		
Constant	−76.218 [−104.591, −47.846]	<0.001
Age (years)	0.327 [0.131–0.522]	0.001
BMI (Kg/m^2^)	0.840 [0.314–1.366]	0.002
Neck circumference (cm)	1.183 [0.363–2.003]	0.005
Scalloped tongue	6.723 [1.002–12.444]	0.022
**ODI (events/hour) Model 2** ^**^		
Constant	−38.250 [−56.605, −19.895]	<0.001
Age (years)	0.349 [0.149–0.549]	<0.001
BMI (Kg/m^2^)	1.126 [0.654–1.599]	<0.001
NC ≥ 40cm + ST	7.557 [1.118–13.996]	0.022

Model 1: R^2^ = 0.272; Durbin-Watson = 1.906; VIF (Variance Inflation Factor) for all independent variables = 1.

Model 2: R^2^ = 0.220; Durbin-Watson = 1.938; VIF for all independent variables = 1.

ODI, oxygen desaturation index; BMI, body mass index; NC, neck circumference; ST, scalloped tongue.

## Discussion

Consistent with previous literature, older age, higher BMI and wider NC were significantly associated with the presence and severity of OSA (Chung et al., 2016). These findings reinforce the importance of physical examination in the initial assessment of patients with suspected OSA.

While ST was not significantly associated with the diagnosis of OSA, it emerged as a relevant predictor of severe OSA. Patients with ST were 3 times more likely to have severe OSA, and its presence significantly increased ODI estimates, which suggests that ST may reflect a greater susceptibility to upper airway collapsibility. These findings support that ST may serve as an accessible clinical marker of the hypoxic burden in OSA patients, particularly when combined with the presence of other clinical signs such as a wider NC. These results align with previous reports suggesting that ST may be indicative of repetitive airway obstruction and pressure, and therefore a physical sign of sustained upper airway compromise, especially in the presence of severe disease (Tomooka et al., 2017).

Interestingly, although ST significantly increased ODI, it did not correlate with T90. This may reflect the distinction between brief, frequent desaturations (ODI), and prolonged periods of oxygen desaturation (T90). Our findings suggest that ST is more closely associated with intermittent hypoxic events rather than sustained desaturation, possibly due to its association with airway resistance rather than prolonged apneic events or nocturnal hypoxemia due to other respiratory diseases. This may also highlight the potential value of ODI over T90 in capturing the episodic nature of hypoxia in severe OSA and the utility of ST as an indirect indicator of this intermittent hypoxic burden.

The combination of a NC ≥ 40 cm and ST indicated a substantial airway compromise, which significantly increased the risk of severe OSA diagnosis by 4.2 times. These results corroborate that the upper airway anatomy is a key factor in the pathophysiology of OSA.

ST assessment is relatively crude and may be subject to interobserver variability. Nevertheless, its main value lies in its simplicity and clinical accessibility, which in conjunction with other clinical signs seems to increase severe OSA suspicion. Macroglossia (true or relative macroglossia (congenital or acquired), and functional macroglossia) and other oral or cranio-facial features, which usually require confirmatory diagnostic procedures (e.g. MRI or CT), and can cause ST, were not assessed, which offers limitations in the establishment of correlations between tongue volume/width, ST and severe OSA (Kim et al., 2014; Scoppa, 2019). Hence, our results should prompt further investigation into the identification of the main contributing factors to ST in patients with severe OSA.

The clinical risk variables included in the STOP-Bang score were not fully included in our database, which did not allow to conclude if ST offers additional predictive value during OSA screening when adjusted to other variables, besides age, BMI, and NC. Adding ST to established screening tools should be investigated and considered, particularly to detect severe OSA.

Selection bias might have contributed to a higher incidence of OSA in this population compared to the general population. Most patients were referred by pulmonologists due to sleep breathing disorder suspicion, leading to an overrepresentation of individuals with OSA. This pre-screened nature of the cohort, combined with the fact that the study was conducted in a single sleep laboratory in Brazil, may limit the generalizability of the observed prevalence of ST and its association with OSA to broader and more diverse populations. ST assessment was performed in a binary manner (present/absent), which is inherently subjective. Although no formal interobserver agreement metrics were calculated, ST assessment was conducted independently by three physicians, and in cases of disagreement, the final classification was based on majority decision, which reduced subjectivity and increased consensus.

Finally, the study did not assess the duration of snoring throughout the night and no information was collected regarding obstruction during REM and NREM sleep. This limited our ability to evaluate the relationship between primary snoring, sleep stage-specific obstruction, and ST. We feel that this should be further investigated in subsequent studies.

## Conclusion

OSA is often overlooked, despite its significant impact on cardiovascular, metabolic, and mental health, as well as its contribution to occupational and motor vehicle accidents that affect public safety (Morsy et al., 2019). Primary care physicians often face heavy workload with limited consultation time (Irving et al., 2017). Thus, it is imperative not only to enhance awareness among primary care physicians regarding the importance of OSA diagnosis and its prevention, but also to provide them with easily accessible tools for the early identification of patients at increased risk of severe OSA, accelerating the start of adequate treatment with positive airway pressure.

Our study suggests that the presence of ST may serve as an additional useful clinical sign of severe OSA among OSA patients. While the evaluation of ST lacks precision and may be subject to interpretation, it remains as a quick and practical tool. Incorporating ST assessment in previously established screening tools that include NC measurement should be considered during OSA screening, particularly severe OSA.

## Data Availability

The original contributions presented in the study are included in the article/supplementary material, further inquiries can be directed to the corresponding author.
